# Case of Typhoid Fever with Unusual Amount of Eruption

**Published:** 1885-05

**Authors:** V. D. Lockhart

**Affiliations:** Homer, Ga.


### CASE OF TYPHOID FEVER WITH UNUSUAL AMOUNT OF ERUPTION.

BY V. D. LOCKHART, M. D., HOMER, GA.

During the summer of 1883, I was called upon to attend a stout, athletic farmer, 32 years of age, suffering with typhoid fever. The attack was mild, and the case presented nothing unusual until the eleventh day. The temperature had not been above 103 deg.; pulse, 100 to 110; slight delirium at night; skin generally moist during afternoons. On the tenth day, noticed usual eruption, several rose-colored spots on surface of chest, disappearing on pressure, and presenting in all respects the usual appearance of the typhoid eruption. But the next morning I found, to my utter surprise and astonishment, that the whole surface of the body and extremities was literally covered by the eruption.

It had also invaded the mucous membrane of the mouth and fauces, and the patient was complaining of a slight soreness in the throat. The face was extremely red and flushed. The spots were so thick on the skin as to almost touch each other, yet each spot was isolated from the rest, very slightly elevated, of a rose or deep pink color, disappearing for an instant on pressure. It was most abundant on the chest and face, where the skin seemed to be somewhat thickened as in measles. It extended both sides, down the thighs, legs, and on the feet, a few spots being seen on the soles of the feet and in the palms of the hands.

There was only one crop of this eruption. For some two or three days, it remained pretty much as I have described. It then faded slowly for a day or two and was again stationary, a larger part of the eruption still remaining nearly a week, when it again began to fade and disappeared during the third week of the fever.

There was no rise of temperature, nausea, diarrhœa or other unfavorable symptom.

The patient rested well, took food moderately well, and recovered in four weeks from commencement of the attack. The severity of the disease was not in any proportion to the eruption, neither did it seem to be in any way modified by it in its general course.

The friends of the patient were very much alarmed, supposing he had measles, scarlet fever or some other disease, instead of typhoid fever, but I have never doubted the correctness of my diagnosis, and am certain he contracted the disease from nursing his brother, who also had typhoid fever at the same time.

The case was one of peculiar interest to me, as I had never seen a case like it before ; and, after examining all the books accessible, have failed to find any report of such case ; therefore, I have thought best to give it to your readers through the medium of your very interesting and valuable journal.

